# Alpha-guaiene isolated from patchouli oil exhibits antifungal activity against four pathogenic fungi

**DOI:** 10.12688/f1000research.154021.1

**Published:** 2025-01-02

**Authors:** Sarifah Nurjanah, Zhaqqu Alhafidz, Maghfira Maulani, Tita Rialita, Elazmanawati Lembong

**Affiliations:** 1 Department of Agricultural and Biosystem Engineering, Faculty of Agro-Industrial Technology, Universitas Padjadjaran, Jatinangor, West Java, 40600, Indonesia; 2 Department of Food Industry Technology, Faculty of Agro-Industrial Technology, Universitas Padjadjaran, Jatinangor, West Java, 40600, Indonesia

**Keywords:** α-guaiene, patchouli oil, antifungal activity, Minimum Inhibitory Concentration, Minimum Fungicidal Concentration

## Abstract

**Background:**

The major component of patchouli oil, patchouli alcohol, is used as fixative and has antimicrobial activity. The function of other components, such as α-guaiene, remains unknown. Therefore, this study reports the antifungal activity of α-guaiene isolated from patchouli oil against for pathogenic fungi: *Aspergillus niger*, *Candida albicans* ATCC 7102, *Microsporum gypseum* ATCC 14683, and *Trichophyton mentagrophytes* ATCC 16404.

**Methods:**

The material from fraction (249°C-254°C) had the highest α-guaiene. Minimum inhibitory concentration (MIC) and minimum fungicidal concentration (MFC) were determined using the microdilution technique to evaluate antifungal activity, with n-hexane and medium serving as negative controls, and ketoconazole and fluconazole serving as positive controls.

**Results:**

The results showed that the MIC value was determined at 45%, 50% for *C. albicans*, 55%, 60% *for A. niger*, 50%, 60% for *M. gypseum*, and 95%, 100% for *T. mentagrophytes*, respectively. Positive and medium controls demonstrated no microbial growth, whereas negative and growth controls revealed the presence of microorganisms. Fungus resistance to α-guaiene *T. mentagrophytes* exhibited the highest MIC value.

**Conclusions:**

Overall, this study reveals that α-guaiene is a promising agent effective against the studied pathogenic fungi.

**List of abbreviations**
PA: Patchouli alcohol
PDA: Potato Dextrose Agar
PDB: Potato Dextrose Broth
RKDU: Riset Kompetensi Dosen UNPAD

## Introduction

Patchouli oil is an essential oil extracted from various plant parts such as flowers, leaves, stems, and roots ( Pandey et al., 2022; van Beek & Joulain, 2018). This oil has promising export potential, as it is regularly used in perfume, soap, pharmaceutical, cosmetic, and other industries. Patchouli oil is renowned for its active constituents and therapeutic benefits ( Leong et al., 2019). Patchouli is a crucial aromatic plant in the perfume industry ( Jain et al., 2022). Furthermore, patchouli oil accounts for approximately 85% of Indonesia’s essential oil exports, with a current annual value of 1,200-1,500 tons. Indonesia exports 90% of the world’s patchouli oil ( Rahmayanti et al., 2018). Notably, patchouli oil’s constituent components, such as patchouli alcohol, α-guaiene, δ-guaiene, α-patchoullene, and seychellene, have several benefits ( Pandey et al., 2021). Pressure, temperature, reflux ratio, and fractionation column all play crucial roles in the fractionating process used to separate these constituents ( Almeida et al., 2018; Nurjanah et al., 2020).

Essential oils have antiviral, antiparasitic, antifungal, bactericidal, insecticidal, and nematocidal properties ( Khaledi & Zahani, 2018). They can be used as bactericides and fungicides against various human-infecting fungus and bacterium types. A pathogenic fungus *Candida albicans* causes candidiasis, whereas *Microsporum gypseum* and *Trichophyton mentagrophytes* cause dermatophytosis ( Moskaluk & VandeWoude, 2022). *Aspergillus niger* is another fungal pathogen that affects the respiratory system by causing different diseases such as aspergillosis ( Fiema et al., 2022). Fortunately, essential oils can be developed as antifungal agents for preventing these. Most compounds derived from essential oils are terpenes and their metabolites derivatives ( D’agostino et al., 2019), such as α-guaiene, a sesquiterpene comprising an average of 11% of the total mass ( Orf et al., 2021). Patchouli oil’s main constituents are patchouli alcohol (27.0%-35%), bulnezen (13.0%-21.0%), and α-guaiene (11.0%-16.0%) ( Górski et al., 2021). Terpenes can inhibit protein and DNA synthesis and promote cell rupture in antibiotic-susceptible and antibiotic-resistant bacteria ( Masyita et al., 2022).

The antimicrobial activity of essential oils, notably patchouli oil, has been reported. Patchouli essential oil has been shown to inhibit Malassezia furfur and to be effective as an antimicrobial and anti-inflammatory agents ( Srivastava et al., 2022). Treatment of *C. albicans* and *T mentagrophytes* with patchouli oil fraction 8 containing 55.59% patchouli alcohol content yielded inhabitation zone of 9.24 mm and 7.70 mm in both fungi, respectively ( Setyaningrum et al., 2017).

Patchouli alcohol is the primary antifungal component of patchouli oil and can be used as a fixative in perfume and related industries. After patchouli alcohol extraction, other components such as α-guaiene can increase patchouli oil’s utilization. Studies on α-guaiene antifungal activity are few, necessitating more investigations on this compound. While patchouli alcohol (PA) exhibited antimicrobial properties against pathogenic bacteria ( *Escherichia coli*, *Pseudomonas aeruginosa*, *Bacillus proteus*, *Shigella dysenteriae*, *Typhoid bacillus*, *Staphylococcus aureus*) ( Yang et al., 2013). In addition, PA proved effective in treating some germs that were resistant to antibiotics, such as methicillin-resistant *Staphylococcus aureus* (MRSA) ( Wan et al., 2021). Therefore, this study explores the antifungal activity of fraction 1 patchouli oil containing 38.8% α-guaiene against *C. albicans*, *A. niger*, *M. gypseum*, and *T. mentagrophytes.*

## Methods

### Materials

Patchouli oil was obtained from distiller in Subang, West Java, Indonesia. The nutrient agar culture used was Potato Dextrose Agar (PDA), whereas the liquid medium was Potato Dextrose Broth (PDB). Other chemicals such as 1% BaCl _2_, 1% H _2_SO _4_, sterile distilled water, 70% alcohol, lactophenol cotton blue (LPCB), 0.85% NaCl, ketoconazole, fluconazole, and n-hexane were also utilized. Chemical compound were obtained from Bratachem, while drugs were obtained from Kimia Farma, Bandung, Indonesia.

The instruments used included Spinning Band Distillation System Model 36-100 from B/R Instrument USA, autoclave, Erlenmeyer flask, beaker glass, scotch bottle, Bunsen, ose needle, Petri dish, cuvette, spectrophotometer, micropipette, microplate, fin pipet, microscope, laminar airflow, oven, spatula, and vortex. The study was conducted using a laboratory experimental method with descriptive analysis, with ketoconazole serving as the positive control for all fungi tests, fluconazole for *M. gypseum* and *T. mentagrophytes*, and n-hexane serving the negative control for the four fungi.

### α-guaiene preparation

To obtain the dominant α-guaiene, the sample was fractionated with a pressure of 10 mmHg, column length of 90 cm, and reflux ratio of 20:1. Patchouli oil was divided into five fractions: 1 at 249 ^o^C-254 ^o^C, 2 at 254 ^o^C-259 ^o^C, 3 at 259 ^o^C-264 ^o^C, 4 at 264 ^o^C-269 ^o^C and 5 at 269 ^o^C-274 ^o^C. Fraction 4 was suspected to contain α-guaiene as the most predominant content.

### Gas Chromatography Mass Spectroscopy (GCMS)

GCMS analysis of the sample was carried out on a Agilent ^®^ 6890 GC-MS, equipped with a split-spitless injector, attached to an Agilent HP-5MS capillary column (30 m x 250 μm, 0.25 μm film thickness). The carries gas was helium at a flowrate of 1.0 mL/min, split ratio 400:1, injector temperature was 280 ^o^C, pressure was 10.48 psi. The transfer line was heated to 280 ^o^C. Identification of the oil components was accomplished by comparison of retention times with standard substances.

### Antifungal activity: Microdilution method

The modified microdilution technique was used to determine the antifungal activity of the sample.

Conidia were removed from the agar slant surfaces using sterile 0.85% saline containing 0.1% Tween 20 (vol/vol). In a final amount of 100 μL per well, the conidia suspension was adjusted with sterile 0.85% saline to a concentration of roughly 1.0 × 10.5. For later usage, the inocula were stored at -20°C. To ensure there was no contamination and to confirm the inocula’s validity, dilutions of the inocula were cultivated on the solid MEA.

Using 96-well microtitre plates and a serial dilution approach, the MIC was determined. Fungal inoculums (10 μL) were added to varying volumes of the studied material that had been dissolved in malt extract broth (MEB). The microplates were kept at 28°C for 72 hours. The concentration that totally inhibited fungal growth (MIC) was the lowest concentration at which no growth was discernible. Serial subcultivation of 2 μL onto microtitre plates containing 100 μL of MEB allowed for the determination of the minimal fungicidal concentration (MFC). The MFC, which indicates 99.5% death of the origin inoculum, was the lowest concentration with no discernible growth.

## Results and Discussion

### Characterization of patchouli oil fraction

To obtain the dominant α-guaiene, the patchouli oil was fractionated using a BR Instrument Spinning Band Distillation System Model 36-100 and divided into five fractions. Fraction 4 was suspected to contain α-guaiene as the pre-dominant content. However, based on the GC-MS test results, the compound was higher in fraction 1 than in the others. After fractionation, the fractions were tested for antifungal activity.

**Figure 1.**
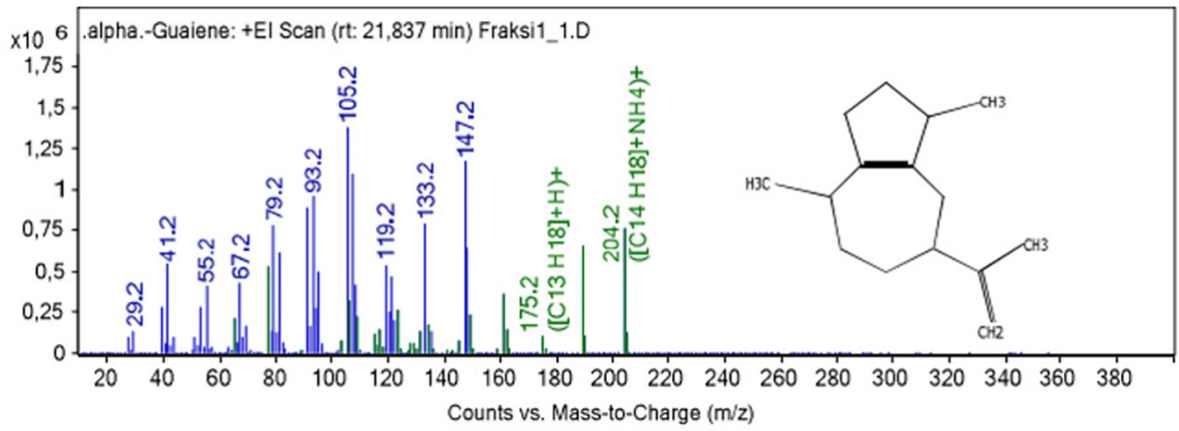
Electron Impact Mass Spectra of α-guaiene.

As determined by GC-MS test conducted on fraction 1 and 4, fraction 1 contained a significantly higher α-guaiene content than fraction 4. The percentage of α-guaiene was detected at peak 2 with retention time at 21.837 min. whereas that of fraction 4 was detected at peak 1 with retention time at 21.293 min. The percentage of α-guaiene content was calculated by dividing the peak area by the total area of the compound formed. Based on these calculations, the percentages in fractions 1 and 4 were 38.8% and 2.1% respectively. Subsequently, fraction 1 patchouli oil was used as the antifungal activity test material. Figure 1 show fragmentation pattern of α-guaiene.

### Fungus identification

**Figure 2.**
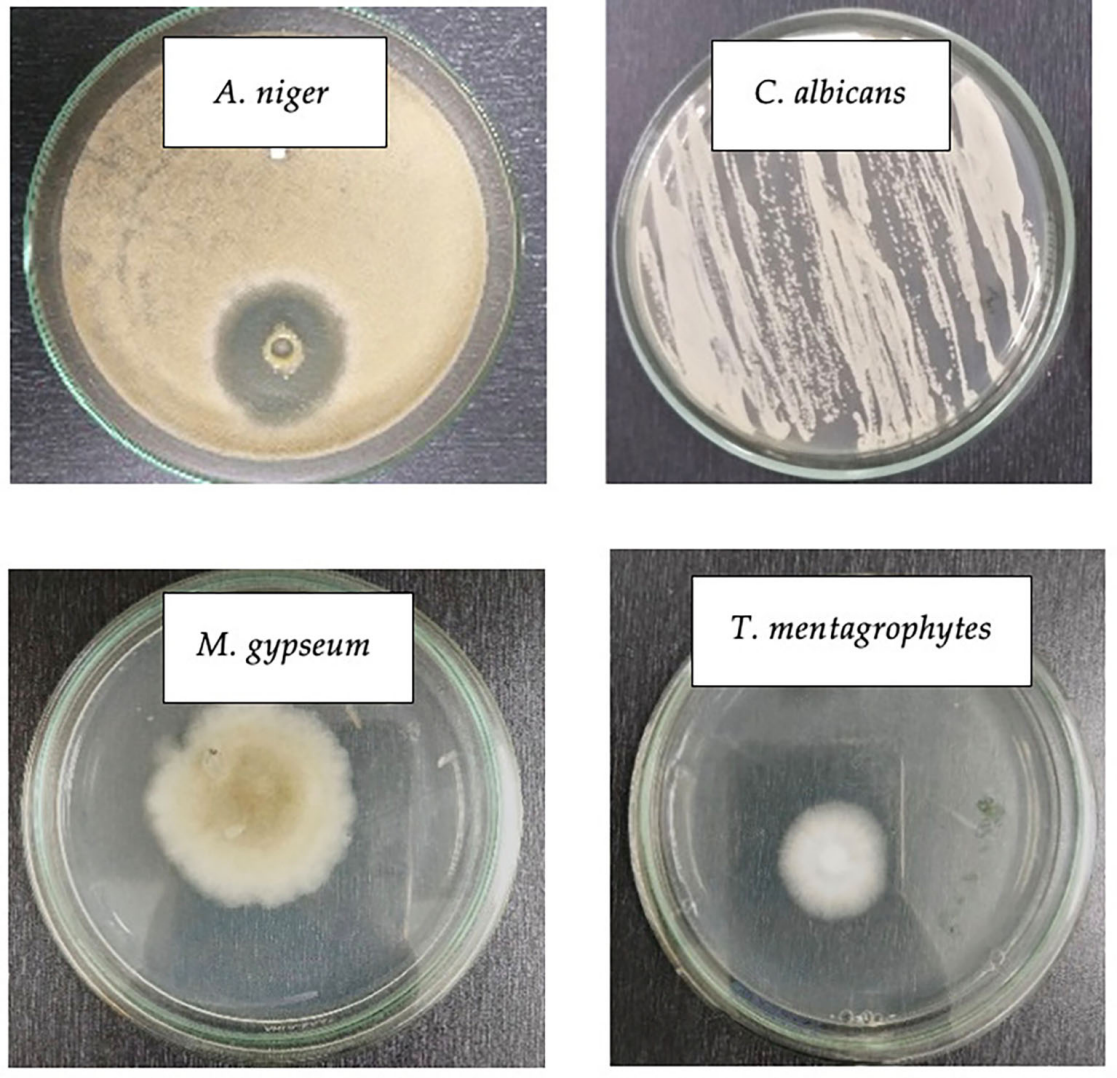
Physical appearance of four pathogenic fungi on PDA medium.

Fungi were macroscopically and microscopically identified. Macroscopic identification involved observation with the eyes in order to directly identify the physical appearance ( Figure 2). Figure 2 (Line 1) show the physical appearance of *C. albicans* and *A. niger* grown on PDA medium. *C. albicans* cultures were white and formed round colonies, corroborating Lee et al’s finding ( Le et al., 2022), that *C. albicans* has a round shape. Allen et al. (2018) reported that *A. niger* culture exhibited white mycelium and brown-black conidia heads. Despite its higher colony size, *A. niger* had less dense colonies than *C. albicans*, which tend to form a firm line based on the groove of the ose needle stroke. In addition, *C. albicans* colonies were spherical, and had a relatively uniform size without hyphae due to their unicellularity, whereas *A. niger* colonies were irregularly spherical, had a nonuniform size, and formed hyphae owing to their multicellularity.

The colony of the fungus suspected to be *M. gypseum* resembled a pile of fine white cotton on top with a little brown powder scattered on the hyphae, corroborating a previous finding ( Putriningsih & Arjentinia, 2018) that *M. gypseum* colonies grew rapidly, were slightly powdery with a blackish-red brown color, and were scattered with a flat surface containing macroconidia.

Macroscopically, although *M. gypseum* and *T. mentagrophytes* did not differ significantly, having white hyphae, *T. mentagrophytes* colonies appeared denser and slightly whiter with a protruding rough or powdery surface corroborating Frías-De-León et al’s findings ( Frías-De-León et al., 2020) that *T. mentagrophytes* colonies were often white to slightly yellowish-white and could sometimes turn violet-red, brown, or pale yellowish with a surface resembling cotton, wax wovwn or granules.

**Figure 3.**
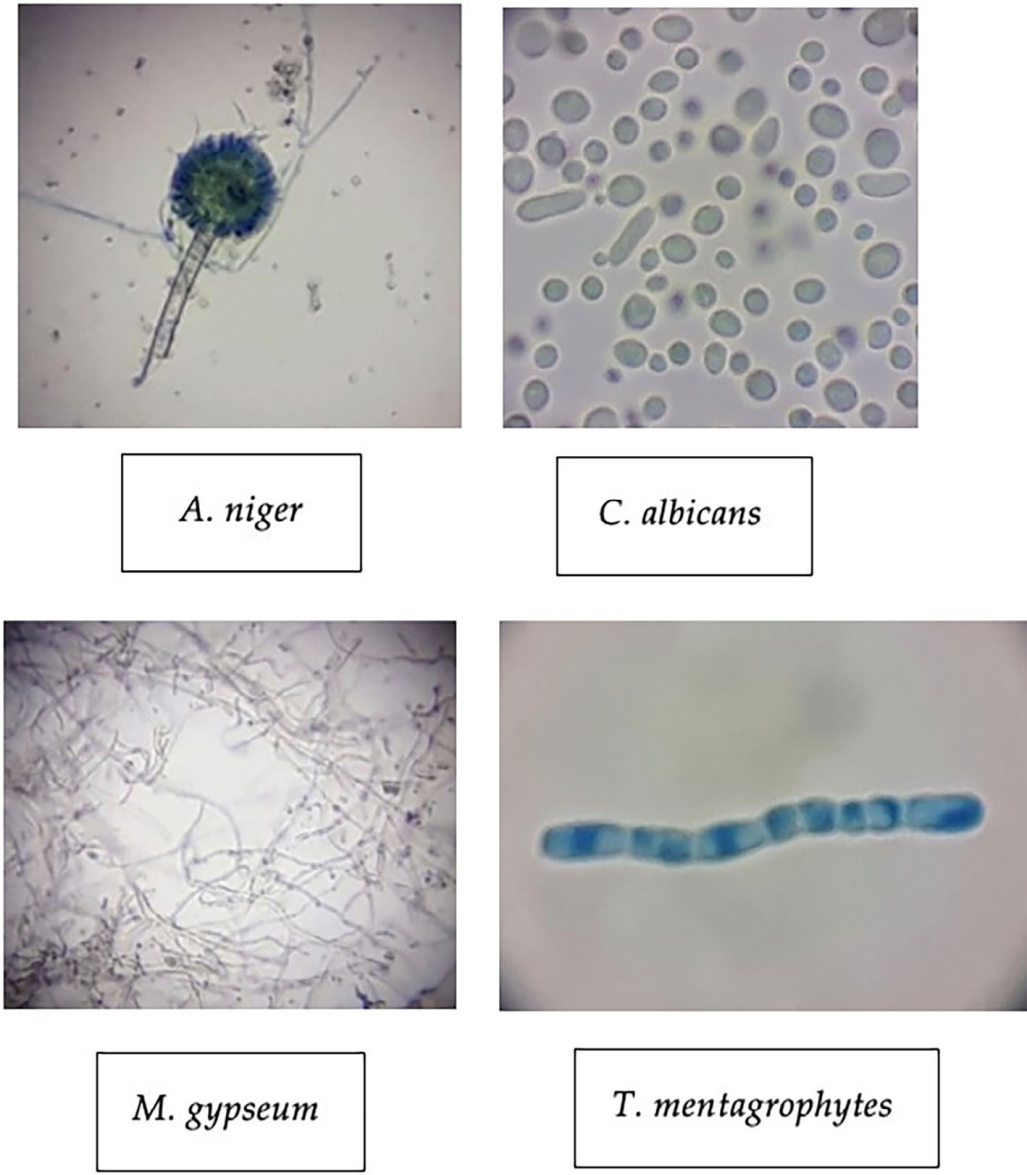
Macroscopic identification of four pathogenic fungi.

The four test fungi were also identified microscopically (1000x) to corroborate their characteristics. *A. niger*, *C. albicans*, *M. gypseum* and *T. mentagrophytes* shared similar characteristics with the same fungi theoretically. Figure 3 showed the identification results of the test. The *A. niger* shows that this fungus has an elongated shape with visible fungal part such as conidia, vesicles, and conidiophores that were obvious by the staining process. Meanwhile, Figure 3 depicting the *C. albicans* compared to *A. niger.* *C. albicans* has a variable round shape with a bluish color and nonuniform cell size. Each cell shows a different individual because this fungus is a unicellular microorganism.

### MIC

**MIC test on *A. niger***

The MIC test results for *A. niger* showed that well with a clear appearance only occurred at 60% concentration treatment, whereas those at 30%, 15%, 7.5% and 3.75% were cloudy with fungal colonies on the surface medium. These observations are relatively weak compared to other studies, such as Yanti et al. (2017) which examined the essential antifungal test on kaffir lime against five type of Aspergillus fungi, revealing an inhibitory mechanism as delayed spore germination and the formation of mycelia at a concentration of 0.05%.

The first observation results showed that the mechanism for fungal growth inhibition occurred only at 60%, indicating that the MIC value was reached at this concentration. Because the range of values between treatments was large, the MIC value may be reach before 60%, specially between 30% and 60%. The second testing process was conducted to minimize concentration different between treatments. Consequently, treatments with new concentrations were made, namely 45%, 50%, 55%, 60% and 65%. A fraction above 60% was intended to predict when the MFC value was not achieved.

As seen in Table 1, the well had a clear appearance before reaching a concentration of 60%, specifically at 55%. This concentration inhibited the growth of *A. niger*, as evidenced by its clear appearance, indicating that fungal growth was inhibited. Because treatment above 55% showed similar results, the MIC value was determined at this concentration.

**Table 1.** MIC observation results in four pathogenic fungi tested.

*A. niger*		*C. albicans*		*M. gypseum*		*T. mentagrophytes*	
Treatments	Appearance	Treatments	Appearance	Treatments	Appearance	Treatments	Appearance
45%	Cloudy	35%	Cloudy	40%	Cloudy	80%	Cloudy
50%	Cloudy	40%	Cloudy	45%	Cloudy	85%	Cloudy
55%	Clear	45%	Clear	50%	Clear	90%	Cloudy
60%	Clear	50%	Clear	55%	Clear	95%	Clear
65%	Clear	155%	Clear	60%	Clear	100%	Clear
Ketoconazole 2%	Clear	Ketoconazole 2%	Clear	Ketoconazole 2%	Clear	Ketoconazole 2%	Clear
				Fluconazole	Clear	Fluconazole	Clear
n-hexane	Cloudy	n-hexane	Cloudy	n-hexane	Cloudy	n-hexane	Cloudy
Growth control	Cloudy	Growth control	Cloudy	Growth control	Cloudy	Growth control	Cloudy
Medium control	Clear	Medium control	Clear	Medium control	Clear	Medium control	Clear

Observation on each control in the second MIC test showed similar results to the first. The positive control of 2% ketoconazole had a clear appearance, indicating that this compound had antifungal activity. The control medium had a clear appearance, showing an absence of fungal or microbial growth, whereas the growth control had a cloudy appearance, indicating that the fungus grew in this treatment. The negative control n-hexane also had a cloudy appearance, suggesting that this compound lacked antifungal activity against *A. niger.*

**MIC test on *C. albicans***

Observation data revealed that none of the treatments produced perfectly transparent wells in C. albicans. In general, a higher concentration implies a clearer well, but at the highest value of 40%, fungal growth was still visible, warranting a re-test to determine perfectly transparent wells. The control treatment on *C. albicans* yielded similar result to the MIC test on *A. niger.* The second test was conducted with treatment concentrations of 35%, 40%, 45%, 50% and 55%. The results showed that a clear appearance began to develop at a concentration of 45%, whereas the treatment at 35% and 45% had a cloudy appearance, suggesting fungal growth. The difference in concentration appeared to have affected the presence of fungi, corroborating a previous study ( Górski et al., 2021) that tested the antifungal activity of patchouli oil at a concentration of 12.5%, 25%, 50% and 100% against *C. albicans.*

Based on the observations, the MIC value was determined at a concentration of 45%, the result obtained was better than that of Kamoda ( Kamoda et al., 2020), stated that the MIC of the red galangal ethanolic extract against the fungus was achieved at 200 mg/mL, but the inhibition only reached at 60%. Differences in antifungal activity for each treatment with varying concentrations also reported by Ningtias et al. (2020), where the MIC value of black garlic extract against *C. albicans* was reached at a concentration of 50%, but active inhibition was fully achieved at 75%.

**MIC test on *M. gypseum***

The MIC observation test on *M. gypseum* fungus with concentration used in twofolds, notably 40%, 20%, 10%, 5% and 2.5% revealed fungal growth in all treatments. Positive and medium controls lacked fungal development, but negative and growth controls demonstrated the opposite. Furthermore, the first observation showed that the highest concentration exhibited no inhibition, although the technique was correctly executed, as indicated by the control treatment, which showed appropriate results. The second test was conducted by increasing the upper limit of concentration and decreasing it with a value range of 5%, hence the new concentration used were 60%, 55%, 50%, 45%, and 40%.

The second MIC test revealed no fugal growth in the wells containing 50%, 55% and 60% of the teat compound, indicating maximum inhibition at these concentrations. The treatments wells overgrown as the number of fungal colonies that grew on the surface increased. Fungal growth was observed at 45% but was not as cloudy as that of 40%, suggesting that a 45% concentration inhibited growth insignificantly. The MIC value is often indicated by the smallest concentration that can inhibit total fungal growth based on visualization, it was determined at 50%.

Positive controls with 2% ketoconazole and fluconazole inhibited fungal growth, as evidence by a clear appearance with a small amount of nonhomogeneous antibiotic precipitate. However, the negative control of n-hexane showed fungal growth, indicating that this compound lacked antifungal activity. The growth control also exhibited fungal growth, suggesting that growth on PDB media was not inhibited. Notably, the control media used was clear, indicating that no undesirable microorganism were present.

**MIC test on *T. mentagrophyhes***

The MIC test results for *T. mentagrophytes* at concentration of 100%, 50%, 25%, 12.5%, and 6.25% of the test compounds showed that only 100% concentration treatment inhibited fungal growth. At 50%, 25%, 12.5% and 6.25% fungal growth remained on the surface, although in varying quantities. The inhibition activity of the test substance at a certain concentration was reflected in the appearance of the media, suggesting the presence or absence of microbes.

In the first test, the MIC value was determined at a concentration of 100%. Because the range of concentrations capable of inhibiting fungal growth was extremely large, the MIC value could be obtained between 50% and 100%. The second test was conducted by minimizing the concentration difference, resulting in values of 100%, 95%, 90%, 85% and 80%.

The second MIC observation test results showed that the wells with 95% and 100% concentration exhibited no fungal or other microbial growth, as indicated by their clear color surface. However, concentration of 90%, 85% and 80% showed varying fungal growth for each treatment. The MIC value is often indicated by the smallest concentration that can inhibit the total growth of the fungi or the appearance of clear media on visualization. Based on the results, the value was determined at 95%.

Because the control treatment for *T. mentagrophytes* yielded identical results to those for *M. gypseum*, the technique used was appropriate. The difference in MIC values demonstrated that fungus resistance to fraction 1 as an antifungal agent differed for each fungal species. *T. mentagrophytes* value exceeded that of *M. gypseum.* These results are directly proportional to the previous inhibition zone test which showed that *T. mentagrophytes* was more resistant than *M. gypseum* to the fraction 1 antifungal agent containing 38.8% α-guaiene

### MFC

The MFC test results for *A. niger*, *C. albicans*, *M. gypseum* and *T. mentagrophytes* revealed fungal growth in wells with a clear medium. Observations were made by creating a new inoculum from only the clear-appearing MIC test results. In order to reduce material requirements and minimize the possibility of errors as wells with cloudy appearance can be ascertained to have fungal growth.

**MFC test on *A. niger***

The MFC test results for *A. niger* shown in Table 2 show that only the 55% concentration of the tree treatments indicated the present of fungal growth. Five of six inoculums created were colonized by fungus. Moreover, the number of fungal colonies in one petri disc was less that 5, this growth was relatively small compared to the original culture without treatment, indicating that the test substance inhibited *A. niger* growth at 55% but did not eradicate this fungus completely. Treatment concentration of 60% and 65% showed no fungal growth in each created inoculum. Based on these results, the MFC value was determined at a concentration of 60%.

**Table 2.** MFC observation results in four pathogenic fungi tested.

*A. niger*		*C. albicans*		*M. gypseum*		*T. mentagrophytes*	
Treatments	Appearance	Treatments	Appearance	Treatments	Appearance	Treatments	Appearance
55%	Cloudy	45%	Cloudy	45%	Cloudy	95%	Cloudy
60%	Clear	50%	Clear	50%	Cloudy	100%	Clear
65%	Clear	55%	Clear	55%	Clear		
Ketoconazole 2%	Clear	Ketoconazole 2%	Clear	Ketoconazole 2%	Clear	Ketoconazole 2%	Clear
				Fluconazole	Clear	Fluconazole	Clear
n-hexane	Cloudy	n-hexane	Cloudy	n-hexane	Cloudy	n-hexane	Cloudy
Growth control	Cloudy	Growth control	Cloudy	Growth control	Cloudy	Growth control	Cloudy
Medium control	Clear	Medium control	Clear	Medium control	Clear	Medium control	Clear

The inoculation wells with the control treatment yielded the expected outcomes under the expected conditions. The positive and medium controls lacked fungal growth, whereas the negative and growth controls indicated opposite results. Moreover, the growth control treatment showed the strongest fungal growth due to the absence of effective antifungal effects.

**MFC test on *C. albicans***

The MFC test results for *C. albicans* presented in Table 2 indicated that the 45% treatment exhibited fungal growth compared to the 50% and 55% treatments. Compared to the control treatment, the number of colonies was much lower for the 45% treatment. Based on these observations, the MFC value was determined at a concentration of 50%.

*C. albicans*’s control treatment yielded similar results to those of *A. niger.* However, the positive control with 2% ketoconazole exhibited no fungal growth. Ketoconazole, a commonly used conventional antifungal agent exhibited antifungal activity that was significantly more potent than fraction 1 of patchouli oil. The results indicate that a concentration of at most 2% ketoconazole can eradicate fungi.

**MFC test on *M. gypseum***

MFC test was conducted at three concentrations, notably 50%, 55% and 60% which were found to inhibit fungal growth in the previous MIC test. The results showed that, at 50% fungal growth remained on the PDA media in all petri dishes. At 55%, four petri dishes were still observed to be overgrown with fungus, whereas the other two were clear. The number of colonies form in each petri dish at 50% and 55% did not exceed three, this growth was relatively small, indicating that the antifungal agent only inhibited fungal growth but did not eradicate *M gypseum.* In addition, at 60%, fungal growth was unobserved in all petri dishes; hence, the MFC value was determined at this concentration. The MFC test results for the control treatment were consistent with the prior MIC test. The positive and medium controls exhibited no fungal growth compared to the negative and growth controls.

**MIC test on *T. mentagrophytes***

MFC testing on *T. mentagrophytes* was conducted at two concentrations, notably 95% and 100%, which were found to inhibit fungal growth in the previous MIC test. The results showed that, at 95%, five petri dishes were still overgrown with fungus, whereas one was not. The number of colonies formed was not significantly high; hence, the antifungal agent appeared to only inhibit but not eradicate fungi. At 100%, fungal growth was unobserved in all Petri dishes; hence, the MIC value was determined at this concentration. The control treatment for the MFC test on *T. mentagrophytes* yielded similar results to those of *M. gypseum.* The positive control at 2% ketoconazole and fluconazole did not support fungal growth. This suggests that 2% ketoconazole and fluconazole as conventional antifungal agents, have significantly more potent effects than fraction 1 of patchouli oil.

## Conclusions

Fraction 1 of patchouli oil containing 38.8% α-guaiene exhibited antifungal activity against *A. niger*, *C. albicans*, *M. gypseum* and *T. mentagrophytes.* The MIC values for each fungus were reached at concentrations of 55%, 45%, 50% and 95%, whereas the MFC value were achieved at 60%, 50%, 60% and 100%, respectively. This study demonstrates that α-guaiene is a prospective agent effective against the investigated pathogenic fungus.

## Data Availability

Figshare: Data of MIC and MFC of alpha-guaiene againts four pathogenic fungi, Doi: 10.6084/m9.figshare.27925854.v1 ( Nurjanah et al., 2024). This project contains the following underlying data:
• MIC of four pathogenic fungi.xlsx • MFC of four pathogenic fungi.xlsx Data are available under the terms of the Creative Commons Attribution 4.0 International license (CC-BY 4.0).
