# Supplementary material for: Alpha-guaiene isolated from patchouli oil exhibits antifungal activity against four pathogenic fungi
Source: F1000Res. 2025 Oct 13;14:4. Originally published 2025 Jan 2. [Version 2] doi: 10.12688/f1000research.154021.2 (PMC12572769; doi:10.12688/f1000research.154021.2)
Supplement: Peer review history

# Reviewer report: Reviewer response for version 2

**DOI:** 10.5256/f1000research.189216.r423550

**Published:** 2025-11-08

**Authors:** Harlinda Kuspradini

**Affiliations:** 1 Mulawarman University Jl. Ki Hajar Dewantara, East Kalimantan, Indonesia

The revised version shows clear improvements. The authors have addressed the previous comments satisfactorily. I have no further major comments at this stage.

Is the work clearly and accurately presented and does it cite the current literature?

Partly

If applicable, is the statistical analysis and its interpretation appropriate?

Not applicable

Are all the source data underlying the results available to ensure full reproducibility?

Partly

Is the study design appropriate and is the work technically sound?

Partly

Are the conclusions drawn adequately supported by the results?

Partly

Are sufficient details of methods and analysis provided to allow replication by others?

Partly

Reviewer Expertise:

Medicinal and Aromatic Plants

I confirm that I have read this submission and believe that I have an appropriate level of expertise to confirm that it is of an acceptable scientific standard.

# Reviewer report: Reviewer response for version 2

**DOI:** 10.5256/f1000research.189216.r424402

**Published:** 2025-10-29

**Authors:** Lal R.K

**Affiliations:** 1 CSIR-Central Institute of Medicinal and Aromatic Plants, Lucknow, India

The manuscript's title renders as "Alpha-guaiene isolated from patchouli oil exhibits antifungal activity against four pathogenic fungi." I have precisely examined this revised research manuscript. The authors have now updated the revised manuscript to include all suggested revisions. The manuscript presents an interesting and relevant study focusing on the isolation and characterization of α-guaiene and its antifungal efficacy. The work contributes to the growing interest in natural bioactive compounds from essential oils as potential alternatives to synthetic fungicides. The study holds potential and addresses an important natural product research area. Finally, the manuscript may be accepted for indexing.

Is the work clearly and accurately presented and does it cite the current literature?

Yes

If applicable, is the statistical analysis and its interpretation appropriate?

Yes

Are all the source data underlying the results available to ensure full reproducibility?

Yes

Is the study design appropriate and is the work technically sound?

Yes

Are the conclusions drawn adequately supported by the results?

Yes

Are sufficient details of methods and analysis provided to allow replication by others?

Yes

Reviewer Expertise:

Plant breeding and Genetic Resource Conservation.

I confirm that I have read this submission and believe that I have an appropriate level of expertise to confirm that it is of an acceptable scientific standard.

# Reviewer report: Reviewer response for version 1

**DOI:** 10.5256/f1000research.168997.r356464

**Published:** 2025-01-16

**Authors:** Harlinda Kuspradini

**Affiliations:** 1 Mulawarman University Jl. Ki Hajar Dewantara, East Kalimantan, Indonesia

The manuscript is well organized but needs improvement.

1. The discussion lacks information on the antifungal mechanism of α-guaiene and does not compare it with patchouli alcohol (PA) or patchouli oil, which would strengthen the argument.
2. The effect of α-guaiene concentration on its antifungal activity is unclear.
3. Include the number of replicates and the statistical analysis method used to validate the results.
4. The method and results sections are inconsistent, particularly in presenting the GC-MS data.
5. The results show that α-guaiene has antifungal properties. It should specify which fungi, α-guaiene is most effective against and discuss its limitations for broader use.
6. Fix some minor inconsistencies in the use of italics for scientific names.

Is the work clearly and accurately presented and does it cite the current literature?

Partly

If applicable, is the statistical analysis and its interpretation appropriate?

Not applicable

Are all the source data underlying the results available to ensure full reproducibility?

Partly

Is the study design appropriate and is the work technically sound?

Partly

Are the conclusions drawn adequately supported by the results?

Partly

Are sufficient details of methods and analysis provided to allow replication by others?

Partly

Reviewer Expertise:

Medicinal and Aromatic Plants

I confirm that I have read this submission and believe that I have an appropriate level of expertise to confirm that it is of an acceptable scientific standard, however I have significant reservations, as outlined above.

# Response

**Published:** 2025-10-02

**Authors:** Sarifah Nurjanah

**Affiliations:** Universitas Padjadjaran, Indonesia

1.Thank you for your valuable feedback. We appreciate your suggestion to improve the discussion section.

 In response, we have added a brief explanation of the antifungal mechanism of **α-guaiene**, highlighting its potential interaction with the fungal cell membrane and its role in disrupting ergosterol biosynthesis, similar to other sesquiterpenes. Furthermore, we have included a comparative discussion between α-guaiene, **patchouli alcohol (PA)**, and **patchouli oil** based on available antifungal activity data from recent studies. This comparison is intended to strengthen the argument regarding the potential of α-guaiene as an active antifungal component.

 2.Thank you for your constructive comment.

 We agree that clarification on the concentration-dependent antifungal activity of **α-guaiene** is important. To address this, we have added supporting information in the Results and Discussion section regarding the correlation between α-guaiene concentration and its inhibitory effect against the tested fungal strain(s). Where available, we also refer to relevant quantitative data from previous studies that demonstrate dose-dependent responses. These additions aim to provide a clearer understanding of the antifungal behavior of α-guaiene across different concentrations.

 3.Thank you for your valuable suggestion.

 We have now included the number of replicates for each experiment and specified the statistical analysis methods used to validate our results. All experiments were performed in **triplicate (n = 3)**, and the data are expressed as **mean ± standard deviation**. Statistical significance was analyzed using **one-way ANOVA**, followed by **Tukey’s post-hoc test** for multiple comparisons, with a significance level set at *p* < 0.05.

 4.Thank you for your insightful comment.

 We acknowledge the inconsistency between the **Methods** and **Results** sections regarding the presentation of GC-MS data. In response, we have carefully revised the **Methods section** to provide a clearer description of the GC-MS operating conditions, including the type of column used, temperature program, injection volume, and identification criteria (comparison with NIST library and retention indices). Additionally, we have ensured that the compounds reported in the **Results section** match the analytical parameters described, and we have clarified how the major and minor components were selected and quantified.

 5.Thank you for your thoughtful and constructive feedback.

 In response, we have clarified in the **Results and Discussion** section that **α-guaiene exhibited the highest antifungal activity against *Fusarium oxysporum***, as indicated by the largest inhibition zone and lowest MIC value among the tested fungal strains. To address the limitations, we have added a brief discussion noting that while α-guaiene shows strong activity against certain fungi, its effectiveness may vary depending on the fungal species, concentration applied, and formulation conditions. Additionally, we highlighted the need for further studies on its **stability, toxicity, and synergistic interactions** with other components to support its broader application in commercial antifungal formulations.

 6.Thank you for pointing this out.

 We have carefully reviewed the manuscript and corrected all inconsistencies in the formatting of scientific names. All genus and species names have now been italicized in accordance with scientific writing conventions throughout the text, including in the abstract, tables, and figure captions.

# Reviewer report: Reviewer response for version 1

**DOI:** 10.5256/f1000research.168997.r356456

**Published:** 2025-01-07

**Authors:** Irmanida Batubara

**Affiliations:** 1 IPB University, Bogor, Indonesia

The manuscript need to add following:

 1. Please add the background why the guaiene concentration only at 38.8%. What is the important, how if lower or higher concentration of guaiene?

 2. Why the author did not compare the activity with only PA or with the patchouli oil before fractionation.

 3. The author more to clarify the fungi name compare to discuss about the activity.

 4. It is important to explain the mechanism of the sample as antifungi.

 5. In the method for GCMS, the author compare the retention time, but in the result author used MS data. Please make it clear

 6. Please add information how many repetition for each measurement

 7. It is better to make table about MIC and MFC value in one table not prepare the observation result.

 8. Please compare the result with positive control as well

 9. in the abstract only MIC value but have two values?

Is the work clearly and accurately presented and does it cite the current literature?

Partly

If applicable, is the statistical analysis and its interpretation appropriate?

Not applicable

Are all the source data underlying the results available to ensure full reproducibility?

Partly

Is the study design appropriate and is the work technically sound?

Partly

Are the conclusions drawn adequately supported by the results?

Partly

Are sufficient details of methods and analysis provided to allow replication by others?

Partly

Reviewer Expertise:

analytical chemistry

I confirm that I have read this submission and believe that I have an appropriate level of expertise to confirm that it is of an acceptable scientific standard, however I have significant reservations, as outlined above.

# Response

**Published:** 2025-10-02

**Authors:** Sarifah Nurjanah

**Affiliations:** Universitas Padjadjaran, Indonesia

We would like to sincerely thank the reviewer for the valuable comments and constructive suggestions that have significantly improved the quality and clarity of our manuscript. We carefully considered each point and have made the following revisions:

1. Several studies have shown that a-guaiene can be isolated using molecular distillation and spinning band vacuum distillation. However, to obtain pure a-guaiene is still very difficult. Molecular distillation can only isolate guaiene with a purity level of 18.80% while using spinning band distillation can produce a-guaiene with a higher purity of 31.05%. For this reason, this study used a-guaiene with a purity of 38.8%, which was obtained from modifying the previous process conditions using spinning bands vacuum distillation.
2. Antimicrobial activity in patchouli oil with several concentrations of PA has been carried out by the author, so that in this paper the focus is on the antimicrobial component of a-guaiene.
3. All other necessary corrections have been incorporated into the revised version of the manuscript (3-9)
